# Use of a Steerable Sheath for Completely Femoral Access in Branched Endovascular Aortic Repair Compared to Upper Extremity Access

**DOI:** 10.1007/s00270-022-03064-8

**Published:** 2022-04-07

**Authors:** Sven R. Hauck, Wolf Eilenberg, Alexander Kupferthaler, Maximilian Kern, Theresa-Marie Dachs, Alexander Wressnegger, Christoph Neumayer, Christian Loewe, Martin A. Funovics

**Affiliations:** 1 Cardiovascular and Interventional Radiology, Department of Bioimaging and Image-Guided Therapy, Medical University of Vienna, Vienna, Austria; 2 Division of Vascular Surgery, Department of General Surgery, Medical University of Vienna, Vienna, Austria; 3 Department of Diagnostic and Interventional Radiology, Ordensklinikum Linz, Linz, Austria; 4 Department of Radiology, Klinik Floridsdorf, Vienna, Austria; 5 Division of Cardiology, Department of Internal Medicine II, Medical University of Vienna, Vienna, Austria; 6 Johannes Kepler University Linz, Medical Faculty, Linz, Austria

**Keywords:** Femoral access, bEVAR, Steerable sheath, Bridging stent grafts

## Abstract

**Purpose:**

To compare bridging stent graft (BSG) implantation in downward oriented branches in branched endovascular aortic repair (bEVAR), using a commercially available steerable sheath from an exclusively femoral access (TFA) with traditional upper extremity access (UEA).

**Methods:**

In a retrospective cohort study, 7 patients with 19 branches in the TFA cohort received BSG insertion using the Medtronic Heli FX steerable sheath from a femoral access, and 10 patients with 32 branches in the UEA cohort from a brachial approach. Technical success, total intervention time, fluoroscopy time, branch cannulation time, and complication rate were recorded.

**Results:**

Technical success was 19/19 branches in the TFA and 31/32 in the UEA cohort. The mean branch cannulation time was considerably shorter in the TFA group (17 vs. 29 min, *p* = 0.003), and total intervention time tended to be shorter (169 vs. 217 min, *p* = 0.176).

**Conclusion:**

Using a commercially available steerable sheath allowed successful cannulation of all branches in this cohort and was associated with significantly shorter branch cannulation times. Potentially, this technique can lower the stroke and brachial puncture site complication risk as well as reduce total intervention time and radiation dose.

**Level of Evidence:**

2b, retrospective cohort study.

## Introduction

Endovascular repair throughout the aorta has spread toward multiple indications and is experiencing a plethora of new applications, with fenestrated and branched endovascular aneurysm repair (f/bEVAR) for complex aortic pathologies being adopted worldwide over the last decade [1–6]. While fenestrations can usually be catheterized from a femoral approach, downward facing branches are the method of choice to engage visceral vessels originating from aneurysmatic parts of the aorta [7]. These branches traditionally require an axillary or brachial arterial approach for catheterization from above [8–10]. Apart from offering a better sealing between the main body and stentgraft inside the aneurysm, branches also offer more variability to catheterize the visceral arteries, which has led to the introduction of off-the-shelf devices suitable for the majority of patient anatomies [11, 12].

However, the brachial or axillary access is associated with potential complications: published access-associated stroke rates are between 0.7 and 3.7% with a recent meta-analysis reporting 2% [13–15]. Approach from above may sometimes even be prohibitive due to hostile anatomic conditions in the thoracic aorta [16, 17]. In addition, the brachial or axillary approach potentially leads to increased radiation dose, longer operation times and additional access site complications such as hematoma and vascular occlusion [13, 15].

Recently, self-made or pre-manufactured steerable sheaths have been utilized to catheterize downward facing branches from a femoral access [14, 16]. One cohort study reported favorable outcomes for a heterogenous mixture of different physician-modified devices for femoral access in bEVAR, including adjunct techniques such as occlusion balloons, external pull-through wires and sheath-in-sheath constructions [14]. The alternative, commercially available modern large-caliber steerable sheaths have only been mentioned in case reports [17]. The purpose of the present study is to report the comparison of a consecutive patient series using a 16 F steerable sheath for the exclusive catheterization of visceral branches in bEVAR with the traditional, upper approach.

## Methods

### Study Design & Population

**Fig. 1:**
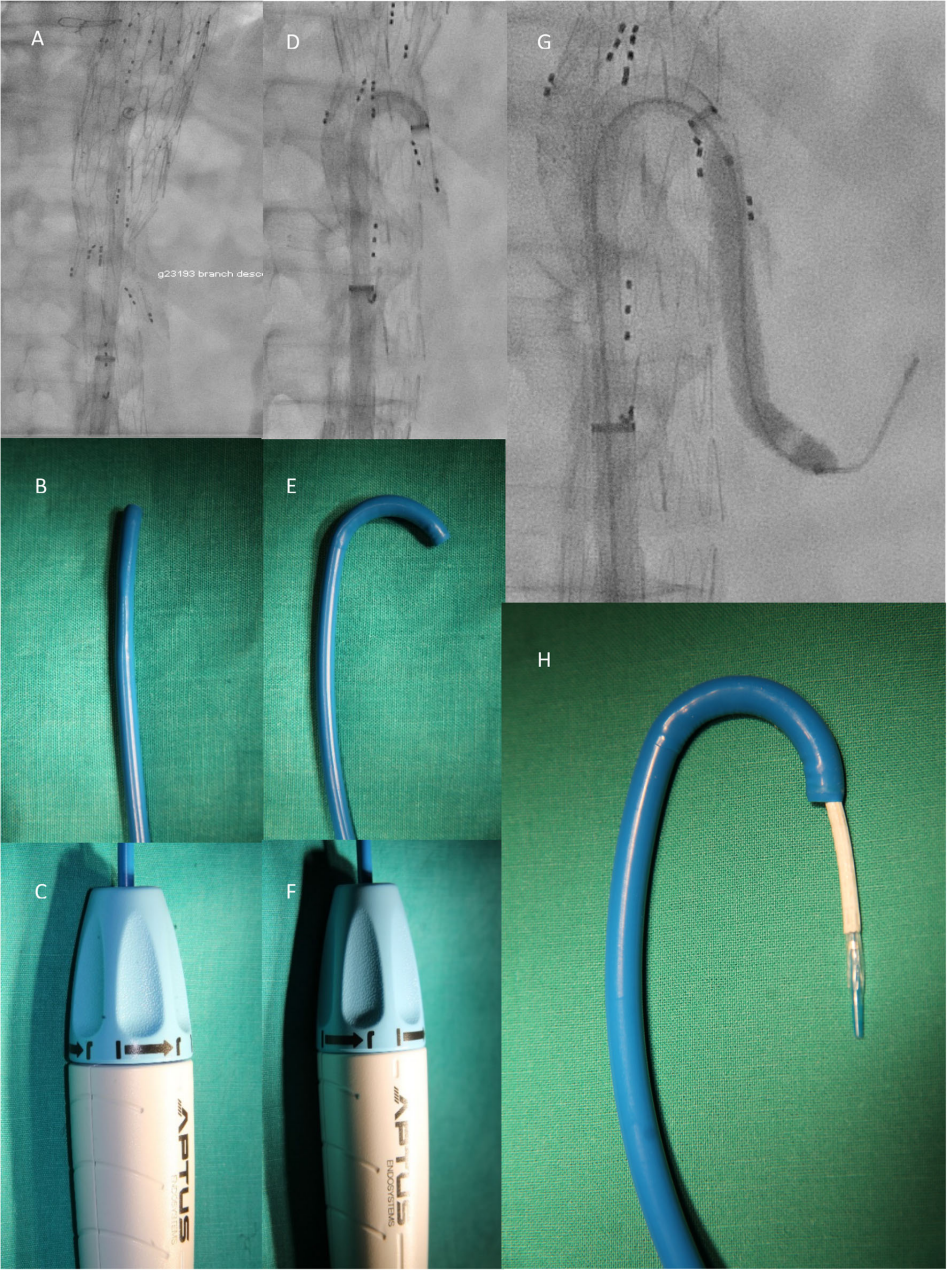
Sheath in stretched position in vivo (**A**), on the bench (**B**) and handle (**C**). Sheath in curved position in vivo (**D**), on the bench (**E**) and handle (**F**). Retrograde BSG deployment via steerable sheath (**G**, **H**)

For this single center, retrospective cohort study, a search of the institutional quality improvement database of endovascular aortic interventions was performed. Late mortality data were obtained through a linkage with the social security system`s death index. Consecutive patients receiving bEVAR between 04/2020 and 06/2021 with at least one downward facing branch and catheterization from a femoral access using a steerable sheath were identified (Fig. 1). Consecutive patients receiving conventional bEVAR in the same timeframe with catheterization from an upper access served as control group. The study was executed under local ethics committee approval, patient consent was waived due to the retrospective nature of the study.

The transfemoral access (TFA) Group (*n* = 7) received exclusively femoral cannulation of the visceral branches using a 16 F steerable sheath (Heli FX Guide, Medtronic, Minneapolis, USA). The upper extremity access (UEA) Group (*n* = 10) received side branch cannulation using a 80–90 cm, 7–8 F sheath (Flexor, Cook Medical, Bloomington, Indiana, USA) inserted via surgical exposure of the axillary or brachial artery. The respective technique used was at the preference of the operator.

### Technique

All procedures were performed in general anesthesia and either surgical exposition of the common femoral artery or percutaneously using suture mediated arterial closure devices (Proglide, Abbott Vascular, Chicago, IL, USA) based on operator preference. Insertion of the BSGs was carried out after deployment of the branched stentgraft (T-branch or CMD device, Cook Medical, Bjaeverskov, Denmark, E-nside, E-xtra, Jotec, Hechingen, Germany) and proximal and distal aortic extensions as required by extent of the disease. Proximal extensions were needed where the required coverage exceeded the maximum length of the branched stentgraft of 200 mm. The Cook T-branch is a multibranched stentgraft of woven polyester sutured to eternal stainless steel Z-stents. The midportion taperes to 18 mm and carries two visceral branches of 8 mm at 12 and 1 o’clock, and two renal branches of 6 mm at 10 and 3 o’clock, respectively, each measuring 18 mm in length. The Jotec multibranch stentgrafts consist of polyester with nitinol stents, the E-xtra carries custom made downward facing branches of 6–8 mm in diameter and 17–19 mm in length, while the E-nside stentgraft carries four inner branches with the branch sutured to the inside of the device and the distal ostium opening not protruding from the exterior surface of the stentgraft, which are precannulated. These inner branches are designed mainly to maneuver in narrow parts of the aorta where catheterization with exterior branches would be compromised. In this study, the choice of main body was based on operator preference.

In the TFA Group, the delivery system was withdrawn to restore femoral perfusion, the pre-applied Proglide sutures were tightened to achieve hemostasis and the 16 F Heli FX Guide was inserted either by itself, or through an additional 20 F sheath, and advanced into the branched stentgraft. The obturator was removed and the tip was deflected via the control handle above the level of the branches and then rotated and retracted until the tip entered the inner ostium of the respective side branch. Using hydrophilic guidewires and catheters the respective renovisceral vessel was catheterized through the branch. After inserting a sufficiently stiff guidewire, 1–2 bridging stentgrafts (BSG) per branch were deployed (VBX, Gore Medical, Flagstaff, AZ, USA: *n* = 25, BeGraft/Plus, Bentley, Hechingen, Germany: *n* = 16, Advanta V12, Getinge, Rastatt, Germany: *n* = 2).

In the UEA Group, a 7–8 F sheath was inserted via a surgical exposure of the left brachial or axillary artery and advanced into the branched stentgraft segment. If stabilization was required the tip of the sheath was held in place using a snare (Amplatz Goose Neck, ev3, Plymouth, MN, USA) from a femoral access. In patients with the E-nside stentgraft, preloaded guidewires through each respective branch could be snared from the UEA and served as stabilization wires during passage through the branch. These guidewires were however not used in the TFA group. Further steps were performed in an analog fashion.

### Outcome

Technical success endpoint was defined as successful deployment of main body and all BSGs and absence of type I or III Endoleak (EL). In addition, fluoroscopy time, contrast agent volume, total intervention time as well as target vessel connection time, defined as the time from first attempt of cannulation to completion angiography of the respective branch after BSG deployment, were recorded.

Secondary outcomes were 30 days adverse events including access site complications, neurologic deficits, unplanned reinterventions, length of procedure-related ICU stay and hospitalization, as well as 30 day and late aortic and all-cause mortality.

### Statistical Analysis

Continuous variables are given as mean ± standard deviation (SD), or median and interquartile range (IQR) as appropriate. Categorical variables are shown as absolute values and prevalence (%) in the respective group. Normal distribution of variables was tested via the Shapiro–Wilk-test and equality of variances with Levene’s test. For binary variables, Fishers exact tests were used. A two-sided independent samples *t*-test with 95% confidence intervals (CI) for normally distributed data, or the Mann–Whitney *U*-test were used for group comparison. Calculations were performed in SPSS 27.0 (IBM, Armonk, NY, USA) at a level of significance of *p* = 0.05.

## Results

### Patient and Procedure Characteristics

The UEA group consisted of 10 patients aged 72.6 (± 10.4) years, with 70% males. The TFA consisted of 7 patients with a mean age of 68.9 (± 10) years, with 85% males. Main comorbidities included hypertension, hyperlipidemia, diabetes mellitus type 2, arterial disease (coronary, peripheral or central), cardiac insufficiency and chronic kidney failure, chronic obstructive pulmonary disease as well as smoking history. All these typical comorbidities were similar between groups. Indication for treatment was thoracoabdominal aneurysm with a mean diameter of 70.8 (± 16.5) mm in the UEA group, and 58.7 (± 6.5) mm in the TFA group (*p* = 0.109). In both groups, two patients had previously undergone aortic surgery or intervention. The intervention was elective in the majority of cases for both groups (*p* = 1.000).

In three cases in the UEA group, the interventions were planned as staged with one side branch cannulation carried out in a later procedure, either due to expected difficulties in catheterization (*n* = 2), to keep anesthesia and operation time short, or in order to provide residual perfusion of the aneurysm sac and the lumbar arteries (*n* = 1) in a patient without CSF drainage. In the UEA group 5 Jotec and 5 Cook stentgrafts, while in the TFA group 1 Jotec and 6 Cook stentgrafts were used. Technical success using the TFA approach was 100%, while in the UEA group there was one side branch catheterization failure and another patient who, despite successful side branch catheterization, died due to multi-organ failure after acute bEVAR in aortic rupture and hemorrhagic shock. In the UEA group there were 3.2 (± 1.1) branches and 1.5 (± 0.7) fenestrations per patient, in contrast to 2.7 (± 0.6) branches and 2.7 (± 1.6) fenestrations per patient in the TFA group. A trend toward shorter intervention times could be seen in the TFA group with 169 (± 47) min compared to 217.4 (± 80.6) min in the UEA group (*p* = 0.176). However, total fluoroscopy time and contrast agent volume were similar (*p* = 0.844; 0.932). Details are included in Table 1.

**Table 1:** Patient, disease and procedure characteristics (BMI body mass index, DM diabetes mellitus, CAD coronary artery disease, COPD chronic obstructive pulmonary disease, CKD chronic kidney failure, PAD peripheral artery disease, CAOD cerebral artery occlusive disease)

	UEA (*n* = 10)	TFA (*n* = 7)	*p*	CI of difference
Age, years	72.6 (± 10.4)	68.9 (± 10)	0.479	− 7.1; 14.4
Sex			0.603	
Male	7	6		
Female	3	1		
BMI	26.6 (± 3.2)	27.6 (± 6.5)	0.669	
Comorbidities				
Hypertension	10	6	0.412	
Hyperlipidemia	9	6	1.000	
DM II	4	2	1.000	
CAD	5	3	1.000	
Smoking	6	4	1.000	
COPD	3	2	1.000	
CKD	2	3	0.593	
PAD	2	2	1.000	
CAOD	4	1	0.338	
Atrial fibrillation	1	2	0.537	
Heart failure	2	0	0.485	
Cancer	1	2	0.537	
Aneurysm type			0.24	
Pararenal	1	1		
Crawford 1	1	1		
Crawford 2 and 3	1	1		
Crawford 4	7	4		
Aneurysm diameter, mm	70.8 (± 16.5)	58.7 (± 6.5)	0.109	
Previous Aortic Surgery/Intervention, *n*	2	2	1.000	
Urgency of intervention			1.000	
Acute	1	1		
Urgent	2	1		
Elective	7	5		
Spinal catheter, *n*	8/10	6/7	1.000	
Intervention time. min	217.4 (± 80.6)	169 (± 47)	0.176	− 24.3; 121.1
Contrast agent, mL	252 (± 70.4)	249.3 (± 51.7)	0.932	− 64; 69.5
Fluoroscopy time, min	110.56 (± 35.6)	107 (± 37.1)	0.844	− 34.4; 41.5
Fenestrations per patient, *n*	1.5 (± 0.7)	2.7 (± 0.6)	0.133	− 3; 0.6
Branches per patient, *n*	3.2 (± 1.1)	2.7 (± 1.6)	0.601	
Staged procedures, *n*	3	0	0.228	
Technical success, *n*			0.485	
Primary	7/10	7/7		
Assisted	8/10			

### Branch Details

In 31 out of 32 branches in the UEA group and in all 19 branches in the TFA group BSGs were successfully deployed (*p* = 0.740). The failure in the UEA group was in one extremely cranially oriented renal artery, where the branch was plugged and a chimney to the renal artery inserted from below. Cannulation time for all branches per patient in the TFA cohort was 46.8 (± 22.5) min and in the UEA cohort 89.9 (± 38.1) min (*p* = 0.017). Mean cannulation time for any branch was 17.2 (± 9.9) min in the the TFA cohort versus 29 (± 14.5) min in the UEA cohort (*p* = 0.003). This effect was present in each target vessel subgroup, with significant differences in celiac artery and SMA as well as a trend in the RRA. See Table 2 for detailed cannulation times.

**Table 2:** Procedural details of side branch cannulation (SMA: superior Mesenteric artery, RRA: right renal artery, LRA: left renal artery)

	UEA branches (*n* = 32)	TFA branches (*n* = 19)	*P*	CI of difference
Successful branches, *n*	31/32	19/19	0.740	
Mean total branch cannulation time per patient	89.9 (± 38.1)	46.8 (± 22.5)	0.017	8.7; 77.5
Mean cannulation time for any branch	29 (± 14.5)	17.2 (± 9.9)	0.003	4.2; 19.3
Mean cannulation time for				
Celiac artery	35.9 (± 15.7)	20.1 (± 7.6)	0.021	2.9; 28.9
SMA	28.9 (± 11.3)	11.8 (± 5.4)	0.016	3.8; 30.4
RRA	22.2 (± 12)	12.3 (± 11.6)	0.219	− 7; 26.7
LRA	26.8 (± 18)	22.6 (± 13.6)	0.610	

### Follow Up and Complications

Due to the recent introduction of the steerable sheath, follow-up was shorter in the TFA group. No statistically significant difference could be found regarding access site complications, post-operative ICU or hospital stay. Technical success, BSG patency, absence of high pressure endoleaks and reintervention rate were similar (Table 3).

**Table 3:** Follow-up variables of patients (ICU, intensive care unit, EL Endoleak)

	UEA (*n* = 10)	TFA (*n* = 7)	*P*	CI of difference
Access site complications, *n*	2	1	1.000	
ICU stay, d	4.9 (± 8.9)	4.7 (± 9.7)	0.475	
Post-op hospital stay, d	19.1 (± 23.4)	12.9 (± 9.3)	0.813	
Follow up clinically, m	5.2 (± 4)	1 (± 1.2)	0.002	
Follow up CT, m	4.7 (± 3.4)	0.7 (± 1.3)	0.010	
Patency of BSG	9/10	7	1.000	
High-pressure EL	1	0	1.000	
Reintervention	2	3	0.593	
Mortality				
Procedure related	0	0	1.000	
Disease related	1*	0		
Unrelated	0	0		

*Death due to multi-organ failure after aortic rupture and acute bEVAR, unsuccessful celiac cannulation, SMA occlusion, multiple reoperations

## Discussion

The main finding of the present study was that using a commercially available steerable sheath allowed for a purely transfemoral access of downward oriented branches in bEVAR in a consecutive series of patients with 100% technical success. Compared to the UEA group, there was a trend to shorter total operation time and branch cannulation time was significantly reduced.

In bEVAR, femoral access offers three distinct advantages: Firstly, brachial access in bEVAR has been associated with an up to 8.4 times increased risk of stroke [18], which can be completely avoided by a transfemoral approach [13, 15]. Secondly, this approach can allow bEVAR in patients that would otherwise be prohibitive due to hostile arch anatomy [16, 17]. Thirdly, the different position of the operator in combination with the reduced operation time may contribute to a reduction in radiation exposure for patient, operator, and team.

Apart from these direct advantages, other studies have reported a lower rate of spinal cord ischemia and a lower rate of thromboembolic events as well as less lower leg ischemia including associated complications when using a transfemoral approach [14, 19, 20]. However, some of these findings are probably rather associated with a concomitant strategy of early removal of the large-caliber sheaths for the main body and exchange for a non-occlusive access to restore femoral blood flow early and minimize ischemia time. In our study most patients have received a spinal drainage, while prophylactic spinal drainage during bEVAR is still controversial.

Other examples of transfemoral bEVAR approach have been reported, among them a series of heterogeneous approaches of physician-modified sheath-in-sheath combinations with wires [14] and two case reports: one describing a successful implantation with the sheath reported here [21] and one using another commercially available steerable sheath (Destino, Oscor, Palm Harbor, USA) [17].

The Heli FX guide has 16F outer diameter and is available in two sizes with 28 mm and 22 mm diameter of the semicircle in the bent position. We used the smaller size due to the fact that the inner diameter of the branched stentgraft segment is 18-22 mm in diameter, depending on the position of the respective branches. Using a 16F access usually allows to restore femoral perfusion once the initial large-diameter sheath of the main body is removed and bleeding controlled by carefully tightening the previously implanted vascular sutures. The relatively large diameter of the sheath and the stability of the curvature allowed us to implant all BSGs without using adjunct techniques such as through-and-through wires or occlusion balloons, and the 16F diameter allowed to advance the BSGs without undue friction even in tight curves. Regarding the different types of BSGs used, no noticeable difference in “pushabillity” was noted between single layer (BeGraft) or double layer type (BeGraft Plus, Advanta V12, VBX). However previous experiments with 7 and 8 F steerable sheaths (TourGuide, Aptus Endosystems/Medtronic, Minneapolis, MN, USA) have shown that in the tight curve inside the branched main body, the BSG expands past its nominal diameter and passage through a tightly curved 7-8F sheath proved impossible. A limitation of the femoral access may be the fact that using an additional sheath with dilatator to enter the visceral artery might be impossible since the secondary sheath would need a caliber of 10–14 F and a length significantly over 90 cm. Nonetheless, all BSGs used in this study entered the visceral artery over a stiff (Amplatz or Rosen) guidewire without further difficulties. The 16 F Heli FX sheath has a European market price of approx. 1900 EUR, which increases total procedure costs. Larger studies are needed to put this cost in relation to potentially lower stroke rates and a lower number of reinterventions.

Another advantage of the relatively large caliber and rigidity of the steerable sheath is the presence of a “force feedback” to the operator. During the careful manipulation of the sheath, the entry of the sheath tip into the branch ostium can be felt as a loss of resistance in the moment when the tip glides from the rim of the branch ostium into the branch lumen. This provides an additional form of information which is unavailable when using standard 6-8F sheaths and 5-6F catheters. Increased contact force using steerable sheaths has already been observed in other endovascular applications, such as cardiac ablation procedures for atrial fibrillation [22, 23].

Strengths of this study include the fact that it represents a cohort study providing a comparative analysis of consecutive patients using brachial or transfemoral access, thereby reducing the potential for selection bias. On the other hand, the study is limited due to the low patient number and by its retrospective nature. However, given the rapid development of techniques in the field of bEVAR, it is likely that we will see widespread adoption of femoral access techniques and consequently larger datasets in the near future [24].

In conclusion, this study demonstrated the feasibility of transfemoral bEVAR using a commercially available steerable sheath, and its efficacy in significantly decreasing branch cannulation time with good technical success rates. These results encourage further use of this method to assess potential advantages of lower stroke rates, access site complications, and radiation burden.
